# Association of the metabolic signature of sleep patterns with breast cancer risk: a prospective cohort study

**DOI:** 10.3389/fendo.2026.1906256

**Published:** 2026-10-15

**Authors:** Qimei Yang, Fang Ai, Yalan Zhao, Zihan Wang, Zhaohong Bi, Rui Ma, Dabao Xu

**Affiliations:** 1 Department of Obstetrics, First People’s Hospital of Yunnan Province, Kunming, Yunnan, China; 2 Department of Gynecology, the Third Xiangya Hospital, Central South University, Changsha, Hunan, China

**Keywords:** breast cancer, genetic susceptibility, metabolic signature, sleep patterns, UK Biobank

## Abstract

**Background:**

Existing evidence regarding the association between sleep patterns and breast cancer development remains inconsistent. This study aimed to investigate the relationship between sleep patterns and breast cancer risk while examining the potential mediating effects of sleep score-related metabolic signatures.

**Methods:**

A total of 220,089 participants from the UK Biobank study were analyzed. Cox proportional hazards models were used to examine the relationships among the sleep score (based on 5 sleep factors), genetic risk (based on 22 variants), metabolic signature (derived from 251 metabolites), and breast cancer incidence, adjusting for age, BMI, sociodemographic, and lifestyle factors. Mediation analysis was used to test whether the metabolic signature mediated the association between the sleep score and breast cancer risk.

**Results:**

A total of 8459 incident breast cancer cases (3.84% of the cohort) were identified during a median follow-up of 11.6 years. Compared with individuals with a sleep score of 5 points, those with a score of 0–1 points had a significantly greater risk of developing breast cancer (HR = 1.30, 95% CI = 1.11–1.52). The sleep-related metabolic signature was also associated with elevated breast cancer risk (HR = 1.62, 95% CI = 1.32–1.99). Mediation analysis revealed that the metabolic signature accounted for 13.5% of the observed association between the sleep score and the incidence of breast cancer.

**Conclusion:**

Maintaining healthy sleep patterns may lower the risk of breast cancer irrespective of genetic predispositions. The metabolic signature partially mediated the association between the sleep score and breast cancer development.

## 1 Introduction

Breast cancer continues to be the most prevalent malignancy in women globally, posing a significant threat to women’s health, as reported by the American Cancer Society (1). Both genetic predispositions and environmental factors, along with lifestyle choices, contribute to the development of breast cancer (2). Emerging evidence suggests that sleep patterns may influence breast cancer risk, although the findings remain inconsistent (3, 4). A Chinese case–control study revealed that a shorter sleep duration was associated with an increased incidence of breast cancer (3), whereas a cross-sectional study reported no significant association (4). Research has focused largely on isolated sleep characteristics—such as sleep duration, insomnia, and daytime napping—rather than comprehensive sleep quality assessment (3–5). Few prospective studies have systematically evaluated how overall sleep patterns influence breast cancer development.

Furthermore, the biological mechanisms linking sleep to breast cancer risk remain incompletely understood. Many studies have shown that genetic susceptibility interacts with sleep behavior to affect health outcomes (6, 7), but how healthy sleep patterns interact with genetic predisposition to influence breast cancer risk remains uncertain. Moreover, sleep critically regulates endocrine function, immune response, and metabolic homeostasis—specifically circadian-regulated metabolic pathways, such as glucose and lipid metabolism— in females, with dysregulation of these systems potentially contributing to breast cancer development through both direct and indirect mechanisms (8, 9). Nevertheless, the mediating effect of metabolic traits on the sleep–breast cancer association has yet to be characterized.

In this large prospective cohort study using data from the UK Biobank study, we aimed to examine the association between healthy sleep scores (based on five sleep factors) and breast cancer risk while also assessing how sleep patterns interact with genetic susceptibility (across low, intermediate, and high-risk groups) to influence breast cancer outcomes. Additionally, we prospectively investigated whether metabolic signatures mediate the relationship between the sleep score and elevated breast cancer risk. We hypothesized that unfavorable sleep patterns are associated with elevated breast cancer risk, that this effect is consistent across different genetic risk groups, and that this association is partially mediated by metabolic signatures.

## 2 Materials and methods

### 2.1 Study population

The UK Biobank study includes a prospective cohort of over 500,000 participants aged 37–73 years who were recruited between 2006 and 2010 (10). Researchers collected comprehensive health data through touchscreen questionnaires, physical measurements, and biological samples. The North West Multi-center Research Ethics Committee granted ethical approval for this initiative. All participants provided written informed consent prior to enrolment.

A total of 273,328 female participants were initially enrolled in this study. 7,126 participants with baseline breast cancer and 46,113 participants with missing data on sleep variables were excluded, including sleep duration (N = 2,491), insomnia (N = 126), chronotype (N = 24,032), snoring (N = 18,896), and sleepiness (N = 568) (**Supplementary Figure 1**). The remaining 220,089 individuals constituted the primary analysis cohort for examining the association between sleep patterns and breast cancer risk. Subsequently, participants without genetic data (N = 5,441) and those of non-European descent (N = 11,076) were further excluded. Ultimately, 203,572 participants were included in the final joint genetic risk and sleep pattern analysis.

### 2.2 Assessment of sleep behavior factors

The participants reported sleep behavior factors data via touchscreen questionnaires (7). Sleep duration was classified as normal (7–8 hours/day), short (<7 hours/day), or long (>8 hours/day). Insomnia symptoms were classified as never/rarely, sometimes, or usually. Snoring responses were recorded dichotomously (no/yes). Chronotype categories were evaluated in four ways: “morning”, “morning” more than “evening”, “evening” more than “morning” and “evening”. Daytime sleepiness was assessed as never/rarely, sometimes, or often/always.

### 2.3 Definition of a healthy sleep score and sleep patterns

A healthy sleep pattern (7) comprised five key sleep behavior factors: an early chronotype (“morning” or “more morning than evening”), a daily sleep duration of 7–8 hours, the absence of frequent insomnia symptoms, the absence of snoring, and minimal daytime sleepiness (“never/rarely” or “sometimes”). These factors were selected based on their established independent and combined associations with cardiometabolic outcomes in previous epidemiological studies (6, 7). Each behavior factor meeting these criteria was scored as 1 point, with nonconforming behavior factors assigned 0 points. The composite score, ranging from 0 to 5 points, reflected overall sleep pattern quality, with higher values indicating healthier sleep. Using this system, we categorized participants into three groups: the healthy (score ≥4), moderate (score of 2–3), and poor (score ≤1) sleep pattern groups.

### 2.4 Definition of the polygenic risk score

In this study, we constructed a polygenic risk score (PRS) for breast cancer based on summary statistics from a published genome-wide association study (GWAS) of breast cancer susceptibility (11). Variants reaching genome-wide significance (P < 5 × 10^-8^) were selected from the GWAS summary statistics and matched to the UK Biobank genotype data (10). Linkage disequilibrium clumping was then performed using a 250-kb window and an r² threshold of 0.10 to obtain approximately independent variants, the details of which are provided in **Supplementary Table S1**. The effect estimates (β) from the GWAS were used to weight the corresponding effect alleles, and the resulting weighted PRS was calculated using PRSice-2 (version 2.3.5) (12), following the analytical framework established in previous studies (6, 7, 13). A total of 22 variants available in our study dataset were retained in the final weighted PRS. Based on the distribution of the PRS, participants were stratified into low (bottom quintile), intermediate (quintiles 2–4), and high (top quintile) genetic risk groups.

### 2.5 Definition of the metabolic signature

To construct the metabolic signature associated with the sleep score, baseline metabolomics data from the UK Biobank were utilized. We assessed the associations between 251 metabolites and the sleep score using elastic net regression, which combines the regularization properties of Lasso and ridge regression to optimize variable selection and enhance model stability. The final metabolic signature score was then calculated as the weighted sum of the metabolites selected by the elastic net model, reflecting their estimated coefficients analyze (14).

### 2.6 Assessment of outcomes

In the UK Biobank study, cancer diagnoses are obtained through linkage to national cancer registries, supplemented by routinely collected healthcare records, including hospital inpatient records. In this study, the main research outcome was incident breast cancer, identified using the International Classification of Diseases, Tenth Revision (ICD-10) code C50 recorded in the linked cancer registry data. The date of the first recorded diagnosis was used as the date of breast cancer occurrence. Participants with breast cancer recorded before baseline were excluded from the analysis.

### 2.7 Covariates

The covariate data originated from baseline physical measurements and touchscreen questionnaire responses. Sociodemographic factors included continuous variables (age, body mass index (BMI), the Townsend Deprivation Index, categorical race data (White/Other), education levels (College or university degree, A/AS levels or equivalent, O levels/GCSEs or equivalent, CSEs or equivalent, NVQ or HND or HNC or equivalent, other professional qualifications and unknown), and employment status (paid employment or self-employed, retired, looking after home and/or family, unable to work because of sickness or disability, unemployment, doing unpaid or voluntary work, full- or part-time student and unknown). Lifestyle factors included smoking status (never, former, current and unknown) and alcohol intake frequency (daily or almost daily, three or four times a week, once or twice a week, one to three times a month, special occasions only, never and unknown).

### 2.8 Statistical analysis

According to the classification of sleep scores, the baseline data of all eligible female participants were statistically analyzed and are reported as the means or percentages. Differences in baseline characteristics across the sleep score groups were evaluated using standardized mean differences (SMDs). The follow-up period was defined as starting from the baseline date and ending on the breast cancer diagnosis, death or cut-off date (December 31, 2021), whichever occurred first.

First, the sleep score was the primary exposure of interest. Cox proportional hazards regression models were used to estimate hazard ratios (HRs) and 95% confidence intervals (CIs) for the association between sleep score and incident breast cancer. The models were adjusted for age, BMI, the Townsend Deprivation Index, ethnicity, education level, employment status, smoking history, and alcohol consumption status to account. The associations of individual sleep factors with breast cancer were also examined for potential confounding factors.

Second, the polygenic risk score (PRS) was used as a measure of genetic susceptibility to breast cancer. We examined the association between PRS and breast cancer risk and further evaluated the joint association of sleep score and PRS with breast cancer risk. The interaction between sleep score and PRS was assessed using a likelihood ratio test.

Third, analyze behavior the association of the sleep score and metabolic signature with breast cancer risk was examined using three models. Model 1 was adjusted for age. Model 2 was additionally adjusted for Townsend Deprivation Index, ethnicity, education level, employment, smoking status, and alcohol consumption status. These covariates were selected *a priori* based on previous evidence and their potential associations with both the sleep score and breast cancer risk (7, 13). Model 3 included both the sleep score and metabolic signature to examine their mutually adjusted associations with breast cancer risk. The correlations among between individual metabolites constituting the metabolic signatures and sleep scores were assessed using multivariate linear regression.

Finally, mediation analysis was conducted to investigate whether the metabolic signature or specific metabolites mediated the association between sleep score and breast cancer risk. The mediation effect and its 95% CI were estimated using the product-of-coefficients method, and the proportion mediated was calculated as the ratio of the mediation effect to the total effect.

We also conducted several sensitivity analyses to test the robustness of our research results: excluding variables missing from the covariates; applying the inverse probability weighting (IPW) method to account for potential selection bias arising from missing covariate information, with weights derived from age, BMI, Townsend Deprivation Index, ethnicity, education level, employment status, smoking status, and alcohol consumption status; considering participants with breast cancer within 2 years postbaseline; excluding COVID-19 pandemic period data; incorporation of breast cancer–related factors, excluding participants with breast cancer within 5 years post-baseline, and excluding individual sleep behaviors.

Statistical analysis was conducted using SAS 9.4 and R 4.4.3 software. A two-sided statistical test was used, and significance was defined as P < 0.05.

## 3 Results

### 3.1 Participant characteristics

**Table 1** summarizes the baseline characteristics of all the participants. At the baseline assessment, sleep scores were distributed as follows: 5 (3.1%), 4 (19.6%), 3 (42.6%), 1 (28.1%), and 0–1 (6.6%). Notably, participants with higher sleep scores tended to be younger, have lower BMI values, and were more likely to be nonsmokers.

**Table 1:** Baseline characteristics of the 220,089 participants.

Baseline characteristics	Healthy sleep score					Standardized differences
	5	4	3	2	0-1	
Number of participants, n (%)	6,919 (3.1)	43,227 (19.6)	93,741 (42.6)	61,748 (28.1)	14,454 (6.6)	
Age, mean ± SD, years	54.8 (8.5)	55.3 (8.4)	56.2 (8.1)	56.8 (7.7)	57.0 (7.4)	0.146
BMI, mean ± SD, kg/m^2^	25.8 (4.7)	26.2 (4.7)	26.6 (4.9)	27.8 (5.4)	29.6 (6.1)	0.343
Townsend Deprivation Index, mean ± SD	-1.4 (3.0)	-1.5 (2.9)	-1.5 (2.9)	-1.3 (3.0)	-0.9 (3.2)	0.093
Ethnicity, White, %	92.8	94.6	95.4	94.7	92.9	0.073
Education, %						0.190
College or university degree	37.4	36.3	32.7	27.6	23.4	
A/AS levels or equivalent	11.6	12.2	12.4	11.7	9.9	
O levels/GCSEs or equivalent	21.4	22	23.7	24.3	24.7	
CSEs or equivalent	5	5	5.3	5.8	6.8	
NVQ or HND or HNC or equivalent	4.2	4	4.2	4.9	5.9	
Other professional qualifications	5.8	5.6	5.7	6	5.8	
Missing	14.6	14.9	16	19.7	23.6	
Employment, %						0.220
In paid employment or self-employed	62.9	60.1	56.1	53	48.4	
Retired	28.4	31.2	34.8	36.3	37.4	
Looking after home and/or family	5.1	5.0	4.7	4.7	4.6	
Unable to work because of sickness or disability	0.7	1.0	1.7	3.3	6.9	
Unemployed	0.7	0.9	1.0	1.1	1.2	
Doing unpaid or voluntary work	0.7	0.7	0.6	0.6	0.4	
Full or part-time student	0.5	0.4	0.3	0.3	0.2	
Missing	0.9	0.8	0.8	0.8	0.8	
Smoking status, %						0.186
Never	68.0	63.9	60.0	56.0	51.6	
Former	25.9	29.1	31.8	33.1	34.4	
Current	5.9	6.8	7.9	10.6	13.7	
Missing	0.2	0.2	0.3	0.3	0.3	
Alcohol intake frequency, %						0.123
Daily or almost daily	12.9	15.5	17.0	16.9	15.7	
Three or four times a week	18.8	21.1	22.1	20.6	17.4	
Once or twice a week	26.1	26.8	26.2	25.7	24.8	
One to three times a month	14.1	13.0	12.6	12.9	13.2	
Special occasions only	15.8	14.0	13.6	15	18.2	
Never	12.3	9.6	8.5	8.9	10.7	
Missing	0	0	0	0.1	0	

Standardized mean differences (SMDs) were used to assess baseline differences across sleep score groups. An absolute SMD < 0.1 indicates a negligible difference between groups.

### 3.2 Association between a healthy sleep score and the risk of breast cancer

After a median follow-up of 11.6 years, 8,459 (3.84%) participants developed breast cancer, with a higher incidence observed among those with lower sleep scores (**Table 2**, **Supplementary Figure 2**). In the analysis of the sleep score and breast cancer risk, participants with a score of 4 demonstrated an HR (95% CI) of 1.10 (0.95, 1.26) relative to those with a score of 5. The HR increased to 1.22 (1.06, 1.39) for a score of 3, 1.29 (1.12, 1.48) for a score of 2, and 1.30 (1.11, 1.52) for scores of 0–1, demonstrating an inverse association between sleep score and breast cancer risk (**Table 2**). For the adjusted model, the overall trend remained consistent, although the HR estimates for all groups showed slight attenuation (**Table 2**). Furthermore, after the exclusion of missing covariates, the inverse probability weighting method, the exclusion of participants with breast cancer within 2 years postbaseline, the exclusion of COVID-19 pandemic period data, incorporation of breast cancer–related factors, and the exclusion of participants with breast cancer within 5 years post-baseline, the results of the sensitivity analysis were generally consistent with those of the main analysis (**Supplementary Table 2**). Morely, excluding individual sleep behaviors, the trend of the results remained the same (**Supplementary Table 3**).

**Table 2:** Relative risk of incident breast cancer according to the healthy sleep score.

Outcomes	Healthy sleep score					*P* for trend
	5 (n=6,919)	4 (n=43,227)	3 (n=9,3741)	2 (n=61,748)	0-1 (n=14,454)	
Breast cancer						
Incident case, No.	221	1,512	3,624	2,513	589	
Incident rate (per 1000 person-years)	2.74 (2.39, 3.12)	3.00 (2.85, 3.16)	3.33 (3.22, 3.44)	3.52 (3.38, 3.66)	3.55 (3.27, 3.85)	
Follow-up time, year (mean)	11.7	11.7	11.6	11.6	11.5	
Rate difference (95% CI)	0.00 (reference)	0.26	0.59	0.78	0.81	
Crude HR (95% CI)	1.00 (reference)	1.10 (0.95, 1.26)	1.22 (1.06, 1.39)	1.29 (1.12, 1.48)	1.30 (1.11, 1.52)	<0.001
Adjusted HR^*^	1.00 (reference)	1.07 (0.93, 1.23)	1.14 (1.00, 1.31)	1.18 (1.03, 1.31)	1.16 (1.00, 1.36)	0.001

*Adjusted for age, BMI, Townsend Deprivation Index, ethnicity, education level, employment, smoking status, and alcohol consumption status. HR, hazard ratio.

The associations between sleep behavior and breast cancer risk are shown in **Supplementary Table 4**. Disrupted sleep patterns, including insomnia, snoring, and excessive daytime sleepiness, were associated with elevated breast cancer risk. Individuals with evening chronotypes face greater susceptibility to breast cancer than their morning-oriented counterparts do. When accounting for confounding factors, daytime sleepiness had a minimal effect on breast cancer risk.

### 3.3 Interaction between the sleep pattern and genetic risk and the association with breast cancer risk

Individuals with elevated genetic susceptibility face a substantially increased risk of developing breast cancer (**Supplementary Table 5**). Compared with the low-risk group, the high-risk group presented a 2.63-fold increased risk, which persisted 2.64-fold after model adjustment. With respect to the combined effect of healthy sleep and polygenic risk scores on the incidence of breast cancer, individuals exhibiting moderate and poor sleep patterns in the high-genetic-risk group demonstrated greater breast cancer risk than did individuals with healthy sleep patterns in the low genetic risk group, with HRs of 3.03 (95% CI: 2.61–3.51) and 2.84 (95% CI: 2.30–3.50), respectively (**Figure 1**). However, within each genetic risk stratum, the differences between sleep score categories were modest with overlapping 95% CIs, suggesting a joint association rather than a strong statistical interaction.

**Figure 1:**
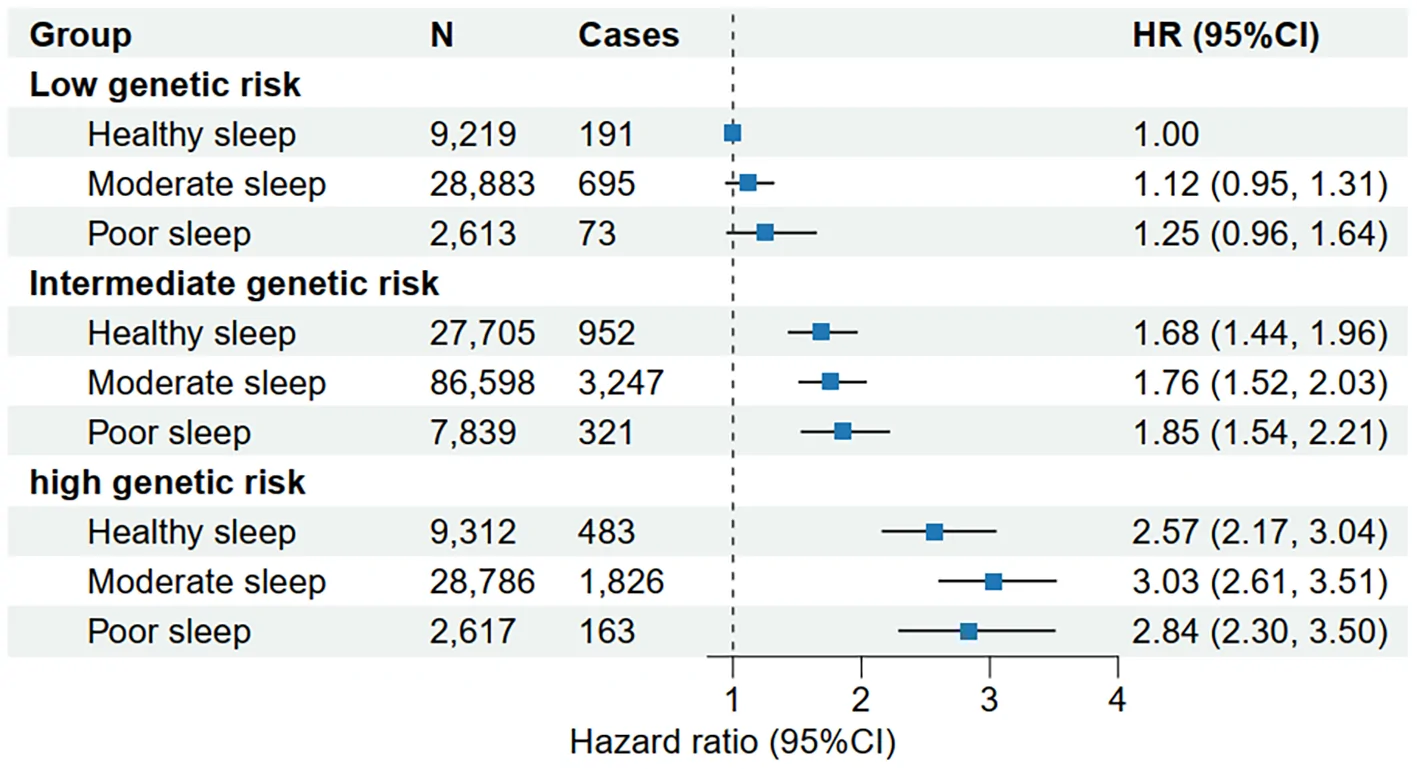
The joint association of genetic risk and sleep pattern with breast cancer incidence.

### 3.4 Mediating effect of the sleep score-related metabolic signature

The metabolic signature associated with the sleep score was also significantly correlated with breast cancer incidence. The HRs were 1.62 (95% CI: 1.32–1.99) for Model 1, 1.64 (1.32–2.02) for Model 2, and 1.53 (1.23–1.90) for Model 3 (**Table 3**). The relationships between sleep score and metabolites, along with intermetabolite correlations, are shown in **Figure 2**. The sleep score was strongly associated with GlycA (elastic net weight ≥0.05), a composite biomarker of systemic inflammation. Moreover, mediation analysis was used to assess how metabolic signatures and metabolites affect the relationship between the sleep score and breast cancer risk. The analysis revealed a significant mediation effect of 0.011 (95% CI: 0.005–0.016) and a direct effect of 0.071 (95% CI: 0.038–0.104), with the metabolic signature mediating 13.5% of the total association (**Figure 3**). Among the metabolites, creatinine, albumin, GlycA, XS_VLDL_PL_pct, L_LDL_C_pct, LA_pct, MUFA_pct, and DHA_pct mediated the association between the sleep score and breast cancer risk (P<0.05) (**Supplementary Table 6**).

**Table 3:** Associations of the sleep score and metabolic signature with the risk of incident breast cancer.

Model	Sleep score		Metabolic signature	
	HR (95% CI)	P value	HR (95% CI)	P value
Breast cancer				
Model 1	1.08 (1.05, 1.12)	<0.001	1.62 (1.32, 1.99)	<0.001
Model 2	1.08 (1.05, 1.12)	<0.001	1.64 (1.32, 2.02)	<0.001
Model 3	1.07 (1.04, 1.11)	<0.001	1.53 (1.23, 1.90)	<0.001

Model 1 was adjusted for age.

Model 2 was further adjusted for age, Townsend Deprivation Index, ethnicity, education level, employment, smoking status, and alcohol consumption status.

Model 3 was a multivariable model that incorporated both the sleep score and metabolic signature for adjustment and was used to explore the independent role of the sleep score and metabolic signatures in breast cancer.

HR, hazard ratio.

**Figure 2:**
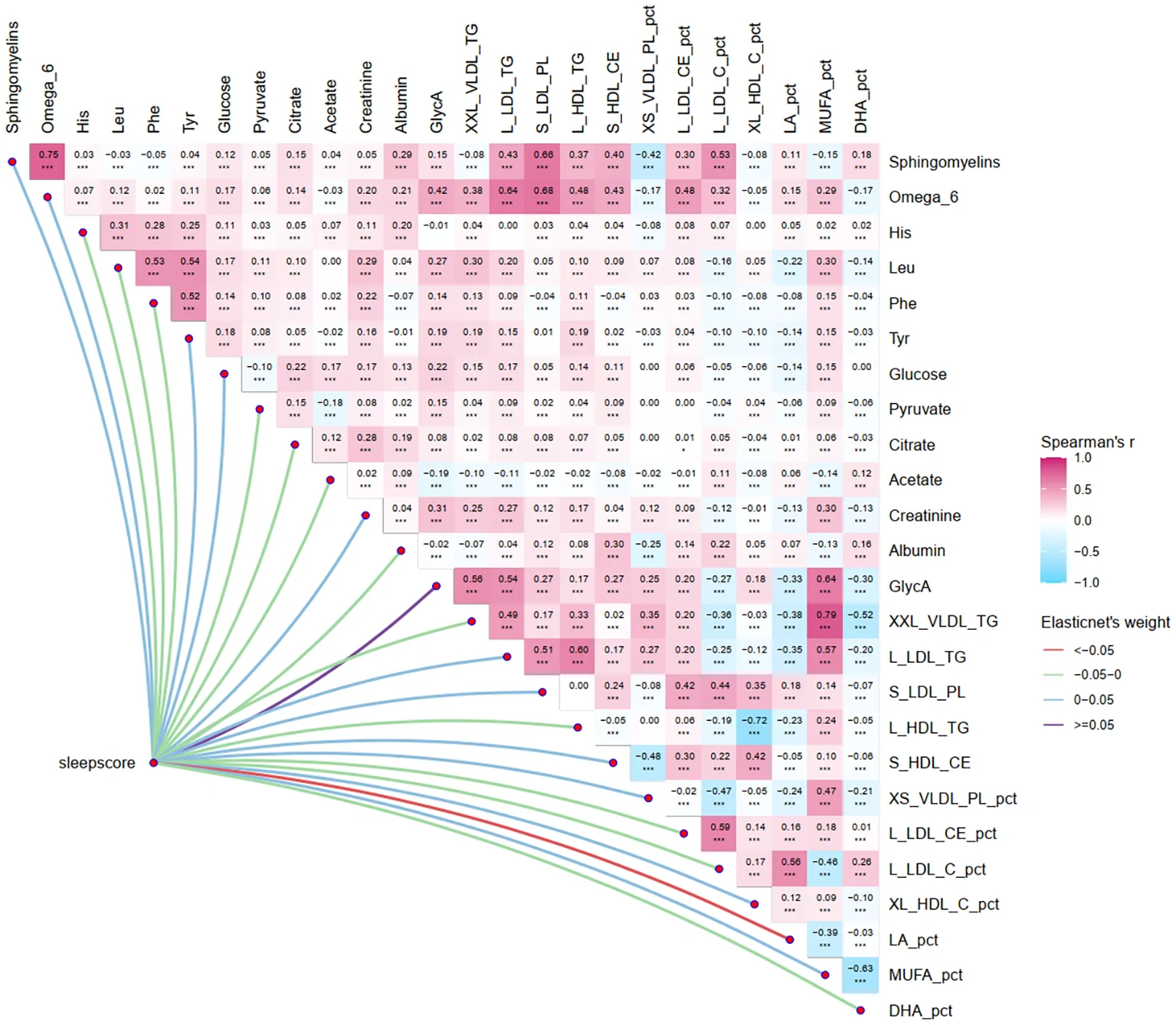
Correlations of the metabolites constituting the metabolic signature and the associations of the metabolic signature with sleep score.

**Figure 3:**
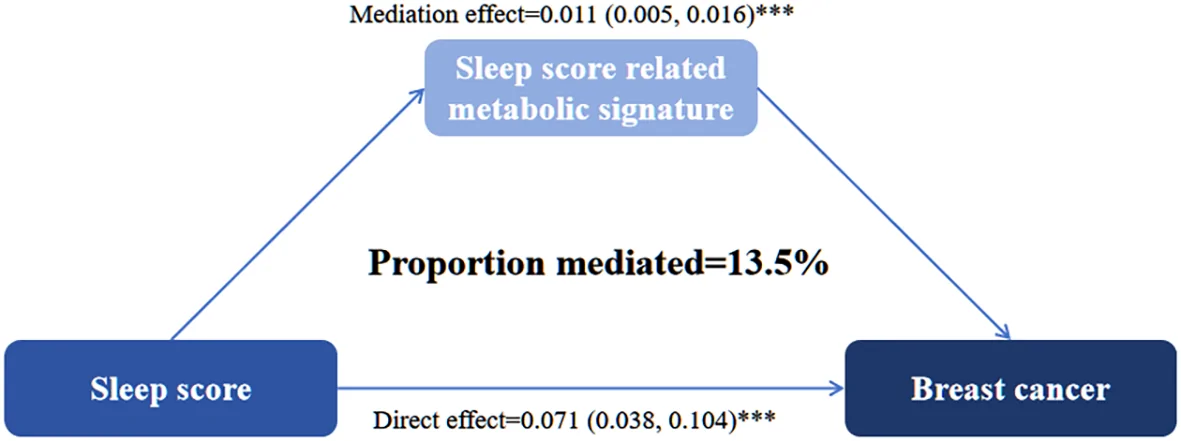
Mediation analysis of the sleep score-related metabolic signature on the relationship between sleep score and breast cancer incidence. *** P<0.001.

## 4 Discussion

This study identified the associations between sleep scores evaluated on the basis of five sleep behavior factors (sleep duration, chronotype, insomnia, snoring, and excessive daytime sleepiness) and the incidence of breast cancer. Lower sleep scores were consistently correlated with elevated breast cancer risk, and the situation persisted across groups at low, intermediate, and high genetic risk. Notably, the sleep score-metabolic signature was also associated with the occurrence of breast cancer and partially mediated the sleep score–breast cancer relationship.

Mendelian randomization studies have demonstrated that sleep patterns affect breast cancer risk (15, 16). Sleep duration and quality are associated with the incidence of breast cancer (4, 17, 18). Here, we developed a composite sleep score incorporating five behavior sleep factors, revealing how combined sleep characteristics collectively affect breast cancer development. This sleep scoring approach has been validated for multiple disease associations (6, 7, 19). Our findings align with existing evidence that a healthy sleep pattern—characterized by early chronotype, a daily sleep duration of 7–8 hours, the absence of frequent insomnia symptoms, the absence of snoring, and minimal daytime sleep—not only decreases breast cancer risk but also mitigates cardiovascular (7) and gastrointestinal disorder risk (6). Various sleep behavior factors interact dynamically, making integrated sleep pattern analysis essential for comprehensively evaluating the influence of sleep on disease development.

For a single sleep behavior factor, the evening chronotype may be an independent risk factor for breast cancer in women (20, 21), with our analysis revealing a 1.1-fold higher risk than that of morning chronotype individuals. Numerous studies have examined the relationship between sleep duration and breast cancer risk, yet their findings remain inconsistent (4, 22, 23). The National Health and Nutrition Examination Survey revealed no significant associations between these variables (4). In contrast, other studies reported an elevated risk of breast cancer for both short and long sleep durations compared a sleep duration of 7–8 hours (22, 23). Similarly, our analysis failed to establish a statistically significant link between sleep duration and breast cancer incidence. The Kailuan cohort study in China demonstrated an association between snoring and elevated breast cancer risk (24), whereas a separate U.S. cohort study reported an inverse relationship (25). Our research revealed that snoring increases the risk of breast cancer. Moreover, women with insomnia appear to face an increased risk of developing breast cancer, a view increasingly supported by current evidence (24, 26, 27). There is little research on daytime sleepiness and breast cancer risk, and our study suggested that excessive daytime sleepiness increased breast cancer risk. Obviously, the inconsistencies between individual sleep behavior and breast cancer risk demonstrate that the sleep scoring method approach offers a more robust foundation for predicting disease risk and designing interventions while overcoming the constraints of analyzing individual sleep factors in isolation.

Furthermore, this study is the first investigation of how sleep patterns interact with genetic susceptibility to influence breast cancer risk. Healthier sleep patterns demonstrated protective effects against breast cancer across all genetic risk strata. Individuals exhibiting both poor sleep quality and high genetic risk require should be prioritized for targeted preventive interventions in breast cancer prevention. Proactive sleep behavior modification may prove beneficial for this high-risk population. Improving sleep quality through sleep behavior-related interventions could effectively mitigate disease risk.

In recent studies, sleep played a critical role in regulating metabolic function, primarily through circadian-driven fluctuations in metabolite levels (28, 29). Insufficient sleep disrupts these metabolic processes and increases disease risk (29, 30), including that of breast cancer (31). The potential mechanisms by which sleep disturbances influence breast cancer risk are multifaceted. Poor sleep increases nocturnal light exposure, which suppresses pineal melatonin secretion, thereby removing its antioxidant and anti-estrogenic protective barriers against DNA damage and endocrine disruption (31–33). Sleep fragmentation also activates pathways including hypocretin/orexin signaling, which is accompanied by glucose metabolism disorders signaling (34). These mechanisms are reflected in the sleep score-related metabolic signature identified in our study. For instance, elevated GlycA within this signature captures the systemic chronic low-grade inflammation induced by sleep fragmentation, which can promote tumor microenvironment remodeling and breast cancer progression (35, 36). Similarly, the identified lipoprotein subfractions and fatty acids (e.g., MUFA, DHA) reflect the endocrine disruption and dyslipidemia mentioned above, which are implicated in breast cancer via lipid peroxidation and hormone synthesis (37). These findings suggest that our metabolic signature is not merely a statistical mediator, but a biological reflection of these sleep-induced mechanisms, serving as a mechanistic link through which sleep influences breast cancer development. To validate and extend these findings, complementary studies are needed. Independent prospective cohorts in diverse populations are essential to confirm generalizability. Furthermore, Mendelian randomization and *in vitro*/*in vivo* experimental models should be employed to strengthen causal inference and elucidate the underlying biological mechanisms by which these metabolites influence breast tumorigenesis.

This study has several advantages. Previous studies have relied primarily on Mendelian analysis and cross-sectional designs to examine the association between sleep and breast cancer risk, whereas our large-scale prospective research design provided more robust epidemiological evidence. Additionally, for the first time, we analyzed the association between the sleep score-related metabolic signature and the risk of breast cancer and identified their mediating regulatory role, which may provide novel strategies for the early screening of breast cancer.

However, several limitations should be acknowledged. First, the study data were drawn from a single national cohort, which may introduce selection bias and limit the generalizability of the findings. Future research should incorporate multinational datasets to strengthen the evidence for the association between sleep patterns and breast cancer risk. Second, despite adjusting for major confounding factors, residual and unmeasured confounding may remain; while additional variables such as hypertension and diabetes may be considered. Third, sleep scores were self-reported and metabolic markers were measured at baseline, which failed to capture dynamic changes and may introduce recall bias. Multiple longitudinal sleep and metabolic marker assessments would enable a more robust analysis of the associations among sleep patterns, metabolic signatures and breast cancer risk. Fourth, the polygenic risk score was constructed from 22 genome-wide significant variants that were available in our dataset. Breast cancer PRSs based on larger variant sets capture more heritability and may provide finer risk stratification.

## 5 Conclusion

In summary, our study demonstrated that a healthy sleep pattern was correlated with reduced breast cancer risk, independent of genetic predisposition. The sleep score-related metabolic signature mediated this association, suggesting that individuals with poor sleep quality could benefit from early breast cancer risk assessment through metabolite screening. Our findings highlight a novel strategy for enhancing early-stage breast cancer prevention.

## Data Availability

The original contributions presented in the study are included in the article/**Supplementary Material**. Further inquiries can be directed to the corresponding author/s.
