# Supplementary material for: Association of the metabolic signature of sleep patterns with breast cancer risk: a prospective cohort study
Source: Front Endocrinol (Lausanne). 2026 Oct 15;17:1906256. doi: 10.3389/fendo.2026.1906256 (PMC13716583; doi:10.3389/fendo.2026.1906256)
Supplement: Supplementary file 1 [file SupplementaryFile1.docx]

**Supplementary materials**

**Association of the metabolic signature of sleep patterns with breast cancer risk: A prospective cohort study**

Qimei Yang, Fang Ai, Yalan Zhao, Zihan Wang, Zhaohong Bi, Rui Ma, Dabao Xu

**Supplementary Figure 1.** **The flowchart of the UK Biobank participants included and excluded in this study.**

**Supplementary Figure 2. Cumulative incidence of breast cancer across different sleep scores.**

**Supplementary Table 1. Genome-wide significant variants for breast cancer.**

| **SNP** | **Chr** | **Position** | **Allele** | **MAF** | **OR (95%CI)** | **P-value** |
| --- | --- | --- | --- | --- | --- | --- |
| rs5776993 | 1 | 110,222,901 | C/CA | 0.12 | 0.95 (0.92-0.96) | 2.6 x 10^-8^ |
| rs9712235 | 2 | 67,881,757 | A/G | 0.26 | 1.04 (1.02-1.05) | 4.8 x 10^-8^ |
| rs4602255 | 2 | 69,392,128 | G/A | 0.45 | 1.04 (1.02-1.05) | 2.0 x 10^-9^ |
| rs1375631 | 3 | 16,778,867 | G/A | 0.5 | 0.97 (0.95-0.98) | 6.8 x 10^-9^ |
| rs2886671 | 3 | 59,373,745 | C/T | 0.43 | 0.97 (0.95-0.98) | 4.3 x 10^-8^ |
| rs34052812 | 3 | 156,535,958 | A/AT | 0.33 | 0.96 (0.95-0.98) | 3.3 x 10^-8^ |
| rs7760611 | 6 | 21,903,533 | C/T | 0.47 | 0.96 (0.95-0.98) | 1.5 x 10^-9^ |
| rs188092014 | 7 | 74,341,926 | G/C | 0.19 | 1.05 (1.03-1.07) | 2.0 x 10^-8^ |
| rs79518236 | 7 | 98,026,554 | ACT/A | 0.23 | 0.96 (0.95-0.97) | 6.6 x 10^-9^ |
| rs142890050 | 8 | 23,480,253 | C/CTT | 0.46 | 0.97 (0.95-0.98) | 3.5 x 10^-8^ |
| rs13256025 | 8 | 25,831,778 | C/T | 0.2 | 1.05 (1.03-1.06) | 1.4 x 10^-8^ |
| rs13277568 | 8 | 116,679,547 | A/G | 0.37 | 0.97 (0.95-0.98) | 2.2 x 10^-8^ |
| rs4742903 | 9 | 106,856,793 | C/G | 0.44 | 0.97 (0.96-0.98) | 2.6 x 10^-8^ |
| rs10838267 | 11 | 44,368,892 | A/G | 0.45 | 0.97 (0.96-0.98) | 4.5 x 10^-8^ |
| 12:29140260 | 12 | 29,140,260 | A/G | 0.09 | 0.93 (0.91-0.95) | 7.7 x 10^-12^ |
| rs11065822 | 12 | 111,600,134 | G/T | 0.37 | 0.96 (0.95-0.98) | 5.9 x 10^-9^ |
| rs1061657 | 12 | 115,108,136 | T/C | 0.26 | 1.04 (1.03-1.06 ) | 2.5 x 10^-10^ |
| rs11652463 | 17 | 70,405,095 | C/G | 0.31 | 0.96 (0.95-0.97) | 4.2 x 10^-8^ |
| rs12962334 | 18 | 20,477,934 | C/G | 0.32 | 1.04 (1.03-1.05) | 3.8 x 10^-9^ |
| rs17743054 | 18 | 42,900,892 | T/C | 0.28 | 0.96 (0.95-0.97) | 1.5 x 10^-10^ |
| rs13039563 | 20 | 52,296,849 | G/A | 0.24 | 1.03-1.06 | 3.1 x 10^-9^ |
| rs9808759 | 21 | 47,780,223 | C/T | 0.07 | 1.07 (1.05-1.09) | 5.8 x 10^-9^ |

SNPs and small indels reaching genome-wide significance (P < 5 × 10⁻⁸) in the source genome-wide association study of breast cancer (reference 11) that were available in the UK Biobank genotype data (reference 10). All 22 variants listed were linkage-disequilibrium clumped (250-kb window, r² < 0.10) and included in the final weighted PRS. MAF, minor allele frequency; OR, odds ratio; CI, confidence interval.

**Supplementary Table 2 Sensitivity analysis of relative risk of incident breast cancer according to healthy sleep score.**

| **Sensitivity analysis** | Healthy sleep score | | | | | ***P* for trend** |
| --- | --- | --- | --- | --- | --- | --- |
|  | 5 | 4 | 3 | 2 | 0-1 |  |
| **Excluding missing variables in covariates** |  |  |  |  |  |  |
| Total, No. | 5,882 | 36,632 | 78,353 | 49,350 | 10,985 |  |
| Incident case, No. | 190 | 1,287 | 2,962 | 1,994 | 439 |  |
| Follow-up time, year (mean) | 11.7 | 11.7 | 11.6 | 11.6 | 11.5 |  |
| Incident rate (per 1000 person-years) | 2.76 (2.38, 3.18) | 3.01 (2.85, 3.18) | 3.25 (3.14, 3.37) | 3.49 (3.34, 3.64) | 3.47 (3.16, 3.81) |  |
| Adjusted HR^*^ | 1.00 (reference) | 1.06 (0.91, 1.23) | 1.11 (0.96, 1.28) | 1.16 (1.00, 1.35) | 1.13 (0.95, 1.34) | 0.008 |
| **Inverse probability weighting method** |  |  |  |  |  |  |
| Total, No. | 6,919 | 43,227 | 93,741 | 61,748 | 14,454 |  |
| Incident case, No. | 221 | 1,512 | 3,624 | 2,513 | 589 |  |
| Follow-up time, year (mean) | 11.7 | 11.7 | 11.6 | 11.6 | 11.5 |  |
| Incident rate (per 1000 person-years) | 2.74 (2.39, 3.12) | 3.00 (2.85, 3.16) | 3.33 (3.22, 3.44) | 3.52 (3.38, 3.66) | 3.55 (3.27, 3.85) |  |
| Adjusted HR^*^ | 1.00 (reference) | 1.07 (0.93, 1.23) | 1.14 (1.00, 1.31) | 1.18 (1.03, 1.35) | 1.16 (1.00, 1.36) | 0.001 |
| **Excluding participants with breast cancer within 2 years post-baseline** |  |  |  |  |  |  |
| Total, No. | 6,882 | 43,015 | 93,191 | 61,372 | 14,362 |  |
| Incident case, No. | 184 | 1,300 | 3,074 | 2,137 | 497 |  |
| Follow-up time, year (mean) | 11.7 | 11.7 | 11.7 | 11.6 | 11.5 |  |
| Incident rate (per 1000 person-years) | 2.28 (1.96, 2.63) | 2.58 (2.44, 2.73) | 2.83 (2.73, 2.93) | 2.99 (2.87, 3.12) | 3.00 (2.74, 3.27) |  |
| Adjusted HR^*^ | 1.00 (reference) | 1.10 (0.95, 1.29) | 1.17 (1.01, 1.36) | 1.21 (1.04, 1.41) | 1.19 (1.00, 1.41) | <0.001 |
| **Excluding the COVID-19 pandemic period data** |  |  |  |  |  |  |
| Total, No. | 6,919 | 43,227 | 93,741 | 61,748 | 14,454 |  |
| Incident case, No. | 205 | 1,409 | 3,379 | 2,317 | 552 |  |
| Follow-up time, year (mean) | 10.7 | 10.7 | 10.7 | 10.6 | 10.6 |  |
| Incident rate (per 1000 person-years) | 2.77 (2.40, 3.18) | 3.05 (2.89, 3.21) | 3.38 (3.27, 3.50) | 3.53 (3.39, 3.68) | 3.62 (3.32, 3.94) |  |
| Adjusted HR^*^ | 1.00 (reference) | 1.07 (0.93, 1.23) | 1.15 (1.00, 1.31) | 1.18 (1.03, 1.36) | 1.17 (1.00, 1.36) | <0.001 |
| **Incorporation of breast cancer–related factors^#^** |  |  |  |  |  |  |
| Total, No. | 6,919 | 43,227 | 93,741 | 61,748 | 14,454 |  |
| Incident case, No. | 221 | 1,512 | 3,624 | 2,513 | 589 |  |
| Follow-up time, year (mean) | 11.7 | 11.7 | 11.6 | 11.6 | 11.5 |  |
| Incident rate (per 1000 person-years) | 2.74 (2.39, 3.12) | 3.00 (2.85, 3.16) | 3.33 (3.22, 3.44) | 3.52 (3.38, 3.66) | 3.55 (3.27, 3.85) |  |
| Adjusted HR^*^ | 1.00 (reference) | 1.07 (0.93, 1.23) | 1.15 (1.00, 1.32) | 1.19 (1.03, 1.36) | 1.17 (1.00, 1.37) | <0.001 |
| **Excluding participants with breast cancer within 5 years post-baseline** |  |  |  |  |  |  |
| Total, No. | 6,830 | 42,612 | 92,222 | 60,765 | 14,218 |  |
| Incident case, No. | 132 | 897 | 2105 | 1530 | 353 |  |
| Follow-up time, year (mean) | 11.8 | 11.8 | 11.8 | 11.7 | 11.6 |  |
| Incident rate (per 1000 person-years) | 1.64 (1.37, 1.94) | 1.79 (1.67, 1.91) | 1.94 (1.86, 2.03) | 2.15 (2.04, 2.26) | 2.14 (1.92, 2.37) |  |
| Adjusted HR^*^ | 1.00 (reference) | 1.06 (0.89, 1.28) | 1.12 (0.94, 1.34) | 1.22 (1.02, 1.45) | 1.18 (0.97, 1.44) | <0.001 |

*Adjust for age, BMI, Townsend Deprivation Index, ethnic, education level, employment, smoking status, and alcohol consumption. HR, Hazards ratio. ^#^Adjust for age, BMI, Townsend index, ethnic, education level, employment, smoking status, alcohol consumption, reproductive history, use of hormone replacement therapy, menopausal status, and most importantly, and family history of breast cancer.

**Supplementary Table 3 Sensitivity analysis of exclusion of individual sleep behaviors.**

| **Sensitivity analysis** | Healthy sleep score | | | | ***P* for trend** |
| --- | --- | --- | --- | --- | --- |
|  | 4 | 3 | 2 | 0-1 |  |
| **Excluding sleep duration** |  |  |  |  |  |
| Total, No. | 8,742 | 59,124 | 112,030 | 40,193 |  |
| Incident case, No. | 277 | 2,117 | 4,326 | 1,739 |  |
| Follow-up time, year (mean) | 11.7 | 11.6 | 11.6 | 11.5 |  |
| Incident rate (per 1000 person-years) | 2.72 (2.41, 3.06) | 3.08 (2.95, 3.21) | 3.33 (3.23, 3.43) | 3.75 (3.58, 3.93) |  |
| Adjusted HR^*^ | 1.00 (reference) | 1.09 (0.96, 1.24) | 1.15 (1.02, 1.30) | 1.24 (1.09, 1.41) | <0.001 |
| **Excluding chronotype** |  |  |  |  |  |
| Total, No. | 28,287 | 104,168 | 70,998 | 16,636 |  |
| Incident case, No. | 979 | 3,943 | 2,871 | 666 |  |
| Follow-up time, year (mean) | 11.6 | 11.6 | 11.6 | 11.5 |  |
| Incident rate (per 1000 person-years) | 2.98 (2.79, 3.17) | 3.25 (3.15, 3.36) | 3.49 (3.37, 3.62) | 3.48 (3.22, 3.76) |  |
| Adjusted HR^*^ | 1.00 (reference) | 1.09 (1.02, 1.17) | 1.14 (1.06, 1.23) | 1.11 (1.00, 1.23) | 0.004 |
| **Excluding insonmia symptoms** |  |  |  |  |  |
| Total, No. | 28,287 | 104,168 | 70,998 | 16,636 |  |
| Incident case, No. | 979 | 3,943 | 2,871 | 666 |  |
| Follow-up time, year (mean) | 11.6 | 11.6 | 11.6 | 11.5 |  |
| Incident rate (per 1000 person-years) | 2.98 (2.79, 3.17) | 3.25 (3.15, 3.36) | 3.49 (3.37, 3.62) | 3.48 (3.22, 3.76) |  |
| Adjusted HR^*^ | 1.00 (reference) | 1.09 (1.02, 1.17) | 1.14 (1.06, 1.23) | 1.11 (1.00, 1.23) | 0.004 |
| **Excluding sleepiness** |  |  |  |  |  |
| Total, No. | 7,017 | 43,896 | 94,879 | 74,297 |  |
| Incident case, No. | 225 | 1,543 | 3,668 | 3,023 |  |
| Follow-up time, year (mean) | 11.7 | 11.7 | 11.6 | 11.6 |  |
| Incident rate (per 1000 person-years) | 2.75 (2.40, 3.13) | 3.02 (2.87, 3.17) | 3.33 (3.22, 3.44) | 3.52 (3.40, 3.65) |  |
| Adjusted HR^*^ | 1.00 (reference) | 1.07 (0.93, 1.23) | 1.14 (0.99, 1.30) | 1.17 (1.02, 1.34) | <0.001 |
| **Excluding snoring** |  |  |  |  |  |
| Total, No. | 9,289 | 55,967 | 110,074 | 44,759 |  |
| Incident case, No. | 294 | 2,056 | 4,358 | 1,751 |  |
| Follow-up time, year (mean) | 11.7 | 11.6 | 11.6 | 11.5 |  |
| Incident rate (per 1000 person-years) | 2.71 (2.41, 3.04) | 3.16 (3.02, 3.30) | 3.41 (3.31, 3.52) | 3.39 (3.23, 3.55) |  |
| Adjusted HR^*^ | 1.00 (reference) | 1.13 (1.00, 1.28) | 1.19 (1.06, 1.34) | 1.17 (1.03, 1.32) | 0.026 |

**Supplementary Table 4 Associations of sleep behaviors and risk of incident breast cancer.**

| **Model** | **N** | **Case** | **Model 1** | |  | **Model 2** | |
| --- | --- | --- | --- | --- | --- | --- | --- |
|  |  |  | **HR (95%CI)** | **P value** |  | **HR (95%CI)** | **P value** |
| **Sleep duration** |  |  |  |  |  |  |  |
| Normal (7-8 hours/day) | 150,717 | 5,812 | 1.00 | - |  | 1.00 | - |
| Short (<7 hours/day) | 17,475 | 709 | 1.06 (0.98, 1.15) | 0.123 |  | 1.03 (0.95, 1.11) | 0.462 |
| Long (>8 hours/day) | 51,897 | 1,938 | 0.97 (0.93, 1.03) | 0.307 |  | 0.96 (0.91, 1.01) | 0.109 |
| **Insomnia symptoms** |  |  |  |  |  |  |  |
| Never/rarely | 42,514 | 1,494 | 1.00 | - |  | 1.00 | - |
| Sometimes | 108,967 | 4,196 | 1.10 (1.04, 1.17) | 0.002 |  | 1.04 (0.98, 1.10) | 0.207 |
| Usually | 68,608 | 2,769 | 1.16 (1.09, 1.23) | <0.001 |  | 1.07 (1.00, 1.14) | 0.049 |
| **Snoring** |  |  |  |  |  |  |  |
| No | 62,479 | 2,595 | 1.00 | - |  | 1.00 | - |
| Yes | 157,610 | 5,864 | 1.12 (1.07, 1.17) | <0.001 |  | 1.07 (1.02, 1.12) | <0.001 |
| **chronotype** |  |  |  |  |  |  |  |
| Morning | 60,478 | 2,204 | 1.00 | - |  | 1.00 | - |
| Morning more than evening | 79,037 | 3,008 | 1.04 (0.99, 1.10) | 0.147 |  | 1.05 (0.99, 1.10) | 0.080 |
| Evening more than morning | 62,051 | 2,510 | 1.11 (1.05, 1.18) | <0.001 |  | 1.12 (1.06, 1.19) | <0.001 |
| Evening | 18,523 | 737 | 1.10 (1.01, 1.19) | <0.001 |  | 1.10 (1.02, 1.20) | 0.022 |
| **Daytime sleepiness** |  |  |  |  |  |  |  |
| Never/rarely | 171,602 | 6,497 | 1.00 | - |  | 1.00 | - |
| Sometimes | 43,061 | 1,744 | 1.08 (1.02, 1.14) | 0.004 |  | 1.03 (0.98, 1.09) | 0.250 |
| Often/all of the time | 5,426 | 218 | 1.11 (1.05, 1.18) | <0.001 |  | 1.05 (0.92, 1.20) | 0.467 |

Model 1 was crude model.

Model 2 was further adjusted for age, BMI, Townsend Deprivation Index, ethnic, education level, employment, smoking status, and alcohol consumption.

**Supplementary Table 5 Relative risk of incident breast cancer according to genetic susceptibility**

| **Outcomes** | | **Genetic susceptibility** | | | ***P* for trend** |
| --- | --- | --- | --- | --- | --- |
|  | **Low genetic risk** | | **Intermediate genetic risk** | **High genetic risk** |  |
| **Breast cancer** |  | |  |  |  |
| N | 40,715 | | 122,142 | 40,715 |  |
| Incident case, No. | 959 | | 4,520 | 2,472 |  |
| Incident rate (per 1000 person-years) | 2.01 (1.89, 2.14) | | 3.18 (3.09, 3.28) | 5.29 (5.08, 5.50) |  |
| Follow-up time, year (mean) | 11.7 | | 11.6 | 11.5 |  |
| Rate difference (95% CI) | 0.00 (reference) | | 1.17 | 3.28 |  |
| Crude HR (95% CI) | 1.00 (reference) | | 1.58 (1.48, 1.70) | 2.63 (2.44, 2.84) | <0.001 |
| Adjusted HR^*^ | 1.00 (reference) | | 1.59 (1.48, 1.70) | 2.64 (2.45, 2.85) | <0.001 |

*Adjust for age and top 10 principal components of ancestry. HR, Hazards ratio.

**Supplementary Table 6 Mediation analysis of sleep score related metabolites on the relationship between sleep score and breast cancer.**

| **Metabolite** | **Total effect** | **Direct effect** | **Indirect effect** | ***P* value** |
| --- | --- | --- | --- | --- |
| Sphingomyelins | 0.082 | 0.082 | 0 (-0.001, 0.001) | 0.896 |
| Omega_6 | 0.082 | 0.082 | 0 (-0.001, 0.001) | 0.780 |
| His | 0.082 | 0.082 | 0 (0, 0) | 0.400 |
| Leu | 0.080 | 0.080 | 0.002 (0, 0.003) | 0.034 |
| Phe | 0.082 | 0.082 | 0 (-0.001, 0.001) | 0.832 |
| Tyr | 0.082 | 0.082 | 0 (-0.001, 0.001) | 0.946 |
| Glucose | 0.082 | 0.082 | 0 (-0.002, 0.001) | 0.748 |
| Pyruvate | 0.082 | 0.082 | 0 (0, 0.001) | 0.080 |
| Citrate | 0.082 | 0.082 | 0 (-0.001, 0.001) | 0.968 |
| Acetate | 0.081 | 0.081 | 0.001 (0, 0.003) | 0.058 |
| Creatinine | 0.080 | 0.080 | 0.002 (0, 0.003) | 0.034 |
| Albumin | 0.081 | 0.081 | 0.001 (0, 0.003) | 0.020 |
| GlycA | 0.076 | 0.076 | 0.006 (0.002, 0.01) | 0.012 |
| XXL_VLDL_TG | 0.080 | 0.080 | 0.002 (-0.001, 0.005) | 0.164 |
| L_LDL_TG | 0.080 | 0.080 | 0.003 (0, 0.005) | 0.046 |
| S_LDL_PL | 0.082 | 0.082 | 0 (-0.001, 0.002) | 0.430 |
| L_HDL_TG | 0.082 | 0.082 | 0 (-0.001, 0) | 0.912 |
| S_HDL_CE | 0.082 | 0.082 | 0 (-0.002, 0.001) | 0.704 |
| XS_VLDL_PL_pct | 0.080 | 0.080 | 0.002 (0, 0.004) | 0.032 |
| L_LDL_CE_pct | 0.082 | 0.082 | 0 (0, 0) | 0.832 |
| L_LDL_C_pct | 0.079 | 0.079 | 0.003 (0, 0.005) | 0.032 |
| XL_HDL_C_pct | 0.082 | 0.082 | 0.001 (-0.001, 0.003) | 0.590 |
| LA_pct | 0.078 | 0.078 | 0.004 (0.001, 0.007) | 0.010 |
| MUFA_pct | 0.075 | 0.075 | 0.007 (0.003, 0.011) | 0.002 |
| DHA_pct | 0.079 | 0.079 | 0.004 (0.001, 0.006) | 0.010 |
